# Can informal social distancing interventions minimize demand for antiviral treatment during a severe pandemic?

**DOI:** 10.1186/1471-2458-13-669

**Published:** 2013-07-18

**Authors:** Amy L Greer

**Affiliations:** 1 Professional Guidelines and Public Health Practice Division, Centre for Communicable Diseases and Infection Control, Public Health Agency of Canada, Ottawa, ON, Canada; 2 Division of Epidemiology, Dalla Lana School of Public Health, University of Toronto, Toronto, ON, Canada

**Keywords:** Antivirals, Behaviour change, Influenza, Mathematical model, Pandemic, Simulation, Social distancing

## Abstract

**Background:**

In the case of a pandemic, individuals may alter their behaviour. A dynamic model incorporating social distancing can provide a mechanism to consider complex scenarios to support decisions regarding antiviral stockpile size while considering uncertainty around behavioural interventions. We have examined the impact of social distancing measures on the demand for limited healthcare resources such as antiviral drugs from a central stockpile during a severe pandemic.

**Methods:**

We used an existing age-structured model for pandemic influenza in Canada and biologically plausible scenarios for severe influenza transmission within the population. We incorporated data from published reports regarding stated intentions to change behaviour during a pandemic as well as the magnitude and duration of time that individuals expected to maintain the behavioural change. We ran simulations for all combinations of parameter values to identify the projected antiviral requirements in each scenario.

**Results:**

With 12 weeks of distancing, the effect is relatively small for the lowest R0 of 1.6 with a projected stockpile to treat 25.6% being required (IQR = 21.7 – 28.7%) unless the proportion of people involved (81%) and magnitude of the behaviour change is large (69% reduction in contacts). If 24 weeks of distancing occurs, with only a low to moderate reduction in contacts (38% or less), it is not possible to bring treatment requirements below 20% regardless of what proportion of the population engages in distancing measures when transmissibility is high (R0 = 2.0; stockpile size = 31%, IQR = 29.2 – 33.5%).

**Conclusions:**

Our results demonstrate that the magnitude and duration of social distancing behaviours during a severe pandemic have an impact on the need for antiviral drugs. However, significant investments over a long period of time (>16 weeks) are required to decrease the need for antiviral treatment to below 10% of the total population for a highly transmissible viral strain (R0 > 1.8). Encouraging individuals to adopt behaviours that decrease their daily contact rate can help to control the spread of the virus until a vaccine becomes available however; relying on these measures to justify stockpiling fewer courses of treatment will not be sufficient in the case of a severe pandemic.

## Background

During an infectious disease outbreak, individuals may change their behaviour in an attempt to protect themselves from becoming infected. Behaviour change is driven by individual perceptions of risk associated with activities such as using public transit or attending public gatherings and may also be influenced by media reporting [1,2]. Changes in behaviour that result in individuals making fewer contacts over the course of a day than would have been the case prior to the behaviour change is one form of social distancing that might occur during a severe influenza pandemic. We define a severe pandemic as one where the new influenza virus transmits easily in the population and causes significant morbidity and mortality.

Non-pharmaceutical public health measures deployed during a pandemic focus on behavioural changes within the population. There has been significant interest in understanding how these changes can influence the projected outcomes of disease transmission models for pandemic influenza [3]. Formal non-pharmaceutical public health measures instituted by public health officials such as school closure decrease the number of contacts between school-aged children. There have been a variety of models that have examined the impact of different school closure strategies on pandemic disease transmission [4-9]. Models that examine the potential impact of multiple public health interventions in combination (e.g. pharmaceutical and non-pharmaceutical interventions) also typically include formal social distancing measures such as school closures [10-12]. Informal social distancing measures such as individual level behaviour change in response to the perceived risk of infection while going about daily activities by changing work routines, or adjusting modes of transportation is another way in which disease transmission during a pandemic may be buffered. More recent models have laid out a general framework for considering this type of informal, dynamic and adaptive behaviour change in models of disease transmission [13-18].

The Canadian government maintains a National Antiviral Stockpile (NAS) in case of an influenza pandemic. Stockpiled drugs are for the direct care of infected patients and not prophylaxis [19]. Before the occurrence of the 2009 influenza A/H1N1 pandemic, Canada used a number of static planning assumptions resulting in plans that were based on an anticipated clinical attack rate of 15-35% [19] resulting in a stockpile that contains enough antivirals to treat 17.5% of the Canadian population. Models that incorporate both social distancing measures and antiviral treatment have primarily examined how to optimally allocate an antiviral stockpile of a pre-defined size using different strategies [20-24]. Alternatively, Kelso et al. [25] have examined how different policies for the diagnosis and treatment of pandemic influenza impact a finite antiviral stockpile over the course of a pandemic. To our knowledge, there are few dynamic models that have been used to understand stockpile size requirements in the face of pandemic influenza uncertainty [26].

We have examined the impact that social distancing behaviour can have on the amount of antiviral treatment required during a severe pandemic. Most developed countries maintain a stockpile of antiviral drugs in the case of a pandemic however, even the largest stockpile is unlikely to contain enough treatment courses to treat more than 20% of a population [19]. In the case of a severe pandemic and in the absence of a sensitive point of care test for pandemic influenza, stockpiles may not be sufficient to meet antiviral demands. In developing countries, where no stockpile exists, behaviour change may be an even more important intervention for minimizing morbidity and mortality. If social distancing were to become a common behaviour during a severe pandemic, one might expect that the disease burden in the population and subsequent healthcare resource needs such as reliance on an antiviral stockpile may be reduced especially if population compliance is high until the point at which a pandemic vaccine becomes available.

The optimal stockpile size will vary depending on the characteristics of the pandemic virus and the care-seeking behaviour of the population. Logistical constraints such as warehousing and the expiry of drugs before they are used are additional considerations. Trade-offs are inevitable and stockpiling for the worst-case scenario is highly cost-prohibitive. The possibility that informal behavioural interventions that don’t rely on school closures will reduce healthcare resource use during a severe pandemic when resources such as antivirals may become thinly stretched is an important consideration for public health decision-makers and pandemic planners. The objective of this model was to evaluate to what extent it might be possible to rely on informal behavioural changes to decrease antiviral treatment needs during a severe pandemic so that less antivirals would need to be held in the National stockpile.

## Methods

### Model structure

We used a previously published, deterministic, SEIR compartmental model to address this research question [27]. The model assumed that all Canadians were in one of several, mutually exclusive health states. Individuals could be susceptible to infection (*S*), or exposed but not yet infectious to others (*E*). In addition, the previously published model was modified to include three different “Infected” compartments. Individuals could be asymptomatically infected (*I*_*A*_), symptomatically infected but never treated with antivirals (*I*_*S*_), or symptomatically infected and treated with antivirals (*I*_*T*_). Lastly, individuals could be recovered from their infection or removed (*R*). We assumed that 40% of all infected individuals were asymptomatic but that asymptomatic and symptomatic individuals were equally infectious [8,28]. Population data used was from the 2006 Canadian Census [29]. The model simulated a 12 month period of model time following the initial introduction of the pandemic influenza strain to Canada.

### Age structure

The model was age-structured (0–4, 5–13, 14–17, 18–23, 24–52, 53–64, 65+) and age-specific mixing patterns were based on empirical data from Mossong et al. [30]. The model was also structured to capture individuals with chronic, underlying medical conditions. The proportion of each age group with at least one chronic condition was estimated from the Canadian Community Health Survey (CCHS) [31]. The model also included a separate pregnant health state (*P*) that represented pregnant women. The population estimated to be in this state at any given point in time was derived from Canadian census data for pregnancies and live births [32,33]. Transitions between compartments were the same for all individuals (Table 1) regardless of chronic disease state or pregnancy however; the probability of receiving vaccine differed by group.

**Table 1:** Parameter values and assumptions used for the Canadian antiviral stockpile model

**Item**	**Strain**	**Value (range)**	**Reference(s)**
Transmissibility			
R0	1957/1958	1.6 (attack rate = 37%)	[8,26,34-36]
	1968/1969	1.8 (attack rate = 41%)	[8,26,34,35,37,38]
	1918	2.0 (attack rate = 45%)	[8,26,34,35,39,40]
Natural history			
Latent period	Seasonal	2.1 days	[41]
Duration of infection	Seasonal	4.8 days	[41]
Pre-existing immunity in individuals > years		0% (0 – 40%)	Assumption
Clinical characteristics			
Proportion symptomatic	1957	60%	[8]
Proportion of symptomatic cases seeking medical attention	1957	70%	[8]
Vaccination			
Vaccine coverage by age group	Age group	RRFSS (%)	
		[27]	
	0-17	60	
	18-22	62	
	23-52	54	
	53-64	65	
	65+	75	

RRFSS – Rapid Risk Factor Surveillance System.

Attack rates are calculated as base case scenarios in the absence of any interventions.

### Pre-existing immunity

In the model, we assume that some proportion of individuals aged 53 and older may be immune to infection by the circulating pandemic strain as a result of previous exposure to a similar influenza strain as seen in past pandemics [42-44]. These individuals are moved to the *R* compartment at time zero. Simulations incorporated a range of pre-existing immunity values from 0% to 40% in 5% increments.

### Influenza transmissibility, natural history and clinical characteristics

We examined a range of basic reproductive numbers (R0) [8,26,34-40,45]. All natural history parameters and ranges examined in the model are outlined in Table 1 and the parameters have been examined in all possible combinations.

### Seasonality

Significant uncertainty exists regarding the time at which a novel pandemic influenza strain may emerge and subsequently spread in Canada. Influenza is not easily transmitted during the northern hemisphere summer likely due to changes in environmental factors (e.g. humidity) which appear to act to decrease influenza transmission [46-48]. Therefore, in scenarios where the introduction of the virus occurs in the spring, the transmissibility of the virus is decreased over the course of the summer and then increased again in the fall when environmental factors become more conducive to influenza transmission (resulting in a relatively small first wave followed by a larger second wave in the fall) [27]. In contrast, when the introduction happens during the fall or winter season, a single large wave is typically seen. These different time scales can significantly affect the observed impact of an intervention strategy such as vaccination. To force seasonality to align with the spring/fall wave phenomenon observed in the northern hemisphere in previous pandemics [46,49], transmissibility was decreased from July to early September. We included two scenarios: a spring/fall scenario with two waves; and a fall/winter scenario with one wave in either the late fall or winter. See [27] for a more detailed description of the implementation of model seasonality.

### Vaccination

We have projected antiviral demand in the presence of vaccination. We assume that vaccine becomes available 6 months after viral emergence. This assumption is based on our experience with vaccine production and distribution capabilities during the 2009 pandemic. We have assumed that it takes 6 weeks to roll out a pandemic vaccination program at the national level. Vaccine efficacy in the model was 70% and it took 10 days for vaccinated individuals to develop vaccine induced immunity [27]. Vaccine prioritization was as follows: 1) pregnant women and all individuals with a chronic underlying condition as defined by the CCHS (regardless of age), 2) healthy children aged 0–4 and healthy adults aged 65+, 3) healthy children aged 5–17, and 4) healthy adults aged 18–64. We assume high vaccine coverage levels as might be expected for a severe pandemic (Table 1) [27].

### Antivirals

In order to receive antivirals in the model, the individual must seek treatment. We include a parameter that describes the proportion of symptomatic cases that seek treatment which is essentially a surrogate for clinical severity (Table 1). We assume that in the absence of a point of care test, all individuals who present for treatment with respiratory symptoms will be eligible to receive antivirals. The model does not include the circulation of other respiratory pathogen so antiviral allocation in the model is for true pandemic cases and not non-influenza cases. The model assumes that all treated individuals receive a 5 day course of antivirals [50]. In the model, antiviral treatment does not decrease transmission to others.

### Social distancing

We have focused on social distancing that impacts the contact rate between individuals in the population (e.g. decreased contact rates = decreased opportunities for influenza transmission). A variety of studies have examined the intention of people to engage in social distancing measures should a severe pandemic occur and we have used these findings to inform our model parameters (Table 2). For the intervention levels that we examined, we did not include a parameter for the waning of compliance over the investigated time period.

**Table 2:** Parameter values and assumptions for implementing social distancing into the model

**Social distancing parameters**			
**Item**	**Experience**	**Value (range examined)**	**Reference**
Proportion of the population *intending* to engage in social distancing	1. Avoiding crowded places	56% (31-81%)	[1,2,51-55]
	2. Avoiding public transportation		
	3. Avoiding public places		
	4. Changing school / work arrangements		
Magnitude of behaviour change	Percent reduction in contacts	38% (7 – 69%)	[13]
Start of social distancing behaviours	Assumption	2 weeks after first imported case	Assumption
Number of weeks that behaviour change is maintained	Assumption	16 weeks (12 – 24 weeks)	[53]
Effect of age on behaviour change	Assumption	No effect	[2,56]

## Results

In the absence of any behaviour change, our model estimates that for a severe pandemic with significant transmissibility (R0 = 2) and no amount of pre-existing immunity in the population, 35.0% of the Canadian population would require access to antivirals from the national antiviral stockpile (IQR = 31.9 – 39.8%). In comparison, a similar pandemic with an R0 equal to 1.8 results in treatment of 32.8% (IQR = 29.0 – 36.7%) and a pandemic with R0 equal to 1.6 results in treatment of 29.4% of the population (IQR = 26.4 – 32.4%).

**Figure 1:**
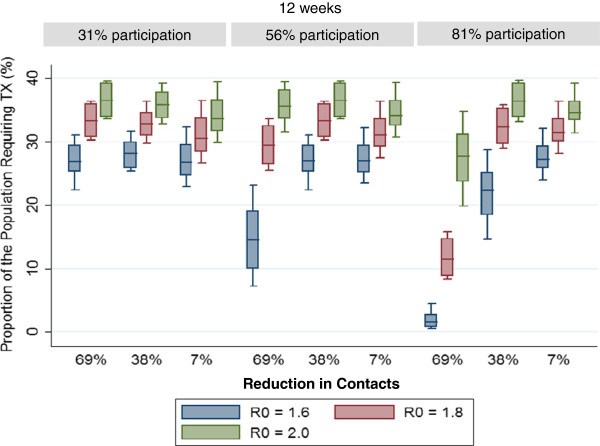
**The median (line within the shaded box), 25**^**th **^**and 75**^**th **^**percentile values (top and bottom of shaded box), and upper and lower adjacent values (error bars) proportion of the Canadian population expected to require antiviral treatment (Y-axis) during a severe pandemic as a function of viral transmissibility and the magnitude of change maintained for 12 weeks.** Each box represents 9 individual simulation runs using a range of pre-existing immunity values from 0-40%. We examined three different levels of behaviour change uptake where between 31% and 81% of the Canadian population engages in social distancing behaviour. The magnitude of the behaviour change ranged from only a 7% reduction in contacts to a 69% reduction in contacts for all scenarios.

**Figure 2:**
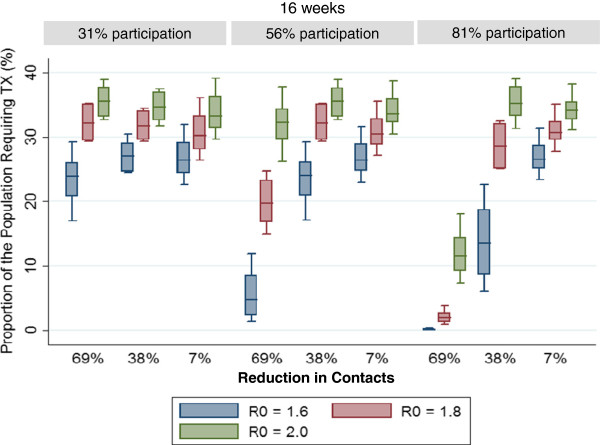
**The median (line within the shaded box), 25**^**th **^**and 75**^**th **^**percentile values (top and bottom of shaded box), and upper and lower adjacent values (error bars) proportion of the Canadian population expected to require antiviral treatment (Y-axis) during a severe pandemic as a function of viral transmissibility and the magnitude of change maintained for 16 weeks.** Each box represents 9 individual simulation runs using a range of pre-existing immunity values from 0-40%. We examined three different levels of behaviour change uptake where between 31% and 81% of the Canadian population engages in social distancing behaviour. The magnitude of the behaviour change ranged from only a 7% reduction in contacts to a 69% reduction in contacts for all scenarios.

**Figure 3:**
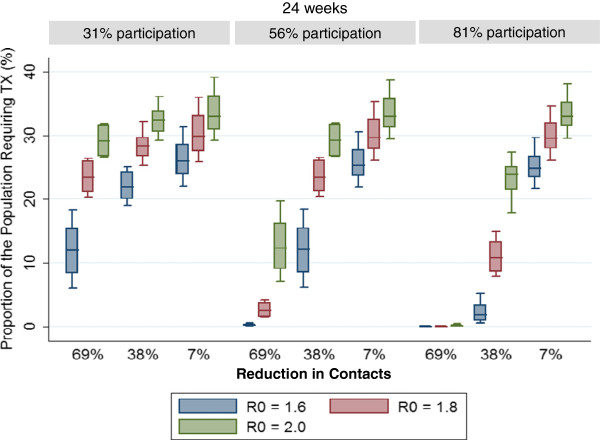
**The median (line within the shaded box), 25th and 75th percentile values (top and bottom of shaded box), and upper and lower adjacent values (error bars) proportion of the Canadian population expected to require antiviral treatment (Y-axis) during a severe pandemic as a function of viral transmissibility and the magnitude of change maintained for 24 weeks.** Each box represents 9 individual simulation runs using a range of pre-existing immunity values from 0-40%. We examined three different levels of behaviour change uptake where between 31% and 81% of the Canadian population engages in social distancing behaviour. The magnitude of the behaviour change ranged from only a 7% reduction in contacts to a 69% reduction in contacts for all scenarios.

The ability of social distancing measures to decrease the number of Canadians requiring antiviral treatment during a severe pandemic depends on the transmissibility of the virus, as well as the willingness, magnitude and duration of distancing measures adopted by individuals. With 12 weeks of social distancing the effect is relatively small even for the lowest R0 of 1.6 (25.6%, IQR = 21.7 – 28.7%) unless the proportion of the population involved (81%) and the magnitude of the behaviour change (69% reduction in contacts) is large (1.4%, IQR = 0.7 – 2.7%) (Figure 1). As the period of social distancing increases to 16 weeks, larger effects can be seen for RO = 1.6 even when the proportion of the population involved is relatively small (23.8%, IQR = 11.9 – 26.6%) (Figure 2). The magnitude of the 16 week effect is most significant for more moderate R0 values (<1.8) (Figure 2). For large R0 values (e.g. 2.0), only a significant investment in social distancing can bring antiviral treatment requirements below 20% (11.5%, IQR = 9.2 – 14.3%). If 24 weeks of distancing occurs, with only a low to moderate reduction in contacts (38% or less), it is not possible to bring treatment requirements below 20% regardless of what proportion of the population engages in distancing measures for high levels of transmissibility (R0 = 2.0; 31%, IQR = 29.2 – 33.5%) (Figure 3). Social distancing measures that are maintained at a moderate to high level for a long period of time (24 weeks) result in very little disease transmission regardless of transmissibility (R0) and therefore antiviral demand from the stockpile is very low (0.006%, IQR = 0.004 – 0.03%) however, the economic impact of such strict measures make a 6 month period of high compliance unlikely (Figure 3).

## Discussion

Our results demonstrate that the impact of social distancing measures on the number of Canadians requiring antiviral treatment during a severe pandemic depends on the transmissibility of the virus, as well as the willingness, magnitude and duration of distancing measures adopted by individuals in the population. We have shown that although informal social distancing measures can reduce the need for antiviral treatment dispensed from a centrally held stockpile, if the pandemic strain were highly transmissible, stockpiles in excess of 20% population coverage could still be required even in the presence of significant behavioural change. Public health planners struggle with the costs and logistics of stockpiling large amounts of treatment. The idea that stockpiling fewer doses and relying on informal social distancing measures in the case of a severe pandemic to decrease demand for the stockpile until vaccine becomes available seems reasonable. However, our results indicate that informal distancing does not actually decrease demand enough to warrant only a small stockpile unless the duration and magnitude of the intervention was sustained for long periods of time with high compliance. Although the main outcome of the model is the quantity of antiviral drugs required from the stockpile to treat pandemic cases, it is also a measure of the use of any health care resources that occur proportionally to the rate at which individuals seek medical attention and could be considered a proxy measure for a variety of healthcare resources that may be in limited supply during a pandemic.

If the duration of behaviour change adopted by the population is maintained for 12 weeks, the impact on the consumption of antivirals is relatively small with a mean of 25.6% (IQR = 21.7 – 28.7%) of the population requiring antivirals from the stockpile. This might be expected in a pandemic where initial media reports highlight the unknown nature of the emerging threat causing fear and panic in the population. However, significant uncertainty exists around the likelihood that compliance would remain high over the course of the 12 weeks. As the situation evolves, people may relax the behavioural changes they had previously implemented if they feel that their risk of infection has decreased [2,57]. Behavioural changes lasting significantly less than 12 weeks are unlikely to have enough of an impact to reduce the need for antivirals from the stockpile which is a finding that is in line with the results of Maharaj and Kleczkowski [15]. Maharaj and Kleczkowski demonstrated that if the disease is highly infectious, social distancing cannot be relied upon to control the epidemic and any social distancing that does occur would have to be extreme in order to have any effect [15].

The duration of time that distancing measures are in place has important implications for the economic stability of a country. In some cases, individuals may be able to change their contact patterns easily for example, by arranging to work from home. However, for individuals who work in an industry that provides critical infrastructure, significantly altering work related contact patterns is likely not a possibility especially if the behaviour change needed to be implemented for up to 6 months. Compliance fatigue would likely play a significant role regardless of if the social distancing were to be maintained for 12 or 24 weeks.

Even though we have assumed high levels of vaccine uptake in the population, vaccination has only a small impact on the overall antiviral stockpile need in the population because of the time at which we assume vaccine becomes available (Table 1). This is especially relevant during a severe pandemic as a virus with a higher R0 value will spread very rapidly and infect many individuals before vaccine becomes available even if vaccine could be produced more quickly. For both in-season and out of season emergence, the 6 month time frame for vaccine availability means that a large number of Canadians will be infected before vaccine is available to them. Reducing the timeframe from viral emergence to vaccine availability would further decrease the demand for antiviral treatment.

Our results are in contrast with a UK study examining antiviral stockpiling. A 2005 study by Gani et al. [26] which modelled antiviral treatment requirements in the UK population suggested that a stockpile size of 12% would be sufficient to treat pandemic cases even if the overall attack rate in the absence of intervention was 25%. However, in this study, the authors assume that treated individuals have a shortened duration of infectiousness which significantly reduces the effective reproductive number as more and more individuals are treated. However, empirical evidence suggests that this is likely only the case if treatment is started within 48 hours of symptom onset [58]. Recent analyses of the 2009 influenza pandemic indicates that in at least one geographic region of Canada, the vast majority of patients receiving antivirals received them > 48 hours after symptom onset [59]. In this case, the effect on subsequent transmission would be negligible and the proposed 12% recommendation would be an underestimate because of suboptimal antiviral treatment timing.

### Limitations

We have not explicitly considered school closure in any of these scenarios nor have we considered behavioural changes such as hand hygiene or coughing into your sleeve as the impact of these at a population level is difficult to quantify. We assume a constant level of behaviour change over some duration of time which is likely unrealistic. Some individuals may fatigue such that the magnitude of their behaviour change wanes over time. Others may react to external factors such as media reports and may increase or decrease the magnitude of their social distancing measures over time based on their perception of risk. A variety of studies have examined the intention of people to engage in social distancing measures should a severe pandemic occur and those are the studies we have used to inform this work. The results of these studies vary significantly especially by geography. For example, Asian populations that experienced SARS tend to respond at higher levels than European populations [51]. Also, there is evidence that the way in which people respond in practice often differs from their response if asked about their “intention” (e.g. healthcare workers intention to vaccinate for seasonal influenza vs. actual vaccination rates) [60,61]. Our model does not provide specific advice for how to distribute the antiviral stockpile. It is possible that in the case of a severe pandemic, stockpiles could be used up relatively quickly. In this case, some of the stockpile should be reserved for treatment of hospitalized cases and other vulnerable groups. Targeted antiviral use in this context would likely be much more effective at preventing deaths per dose however; our model has not specifically examined strategies for optimal deployment or management of a stockpile. The model does not consider the possibility of reduced transmission due to antiviral treatment. There is evidence that individuals who receive treatment within 48 hours of symptom onset are less infectious to others [62]. However, it is unclear what proportion of cases would actually begin treatment within this 48 hour window. During the 2009 H1N1 influenza pandemic in Canada, many cases received antiviral treatment outside of this window [59]. If antiviral treatment does decrease influenza transmission by making treated individuals less infectious to others this could lead to a lower population attack rate, and in turn to lower antiviral usage than we project here [25,62]. We have also not considered antiviral wastage due to the presence of other co-circulating respiratory pathogens although this is an area that has important implications for stockpiling and is an area of ongoing research.

## Conclusions

Using an age-structured model of pandemic influenza transmission in the Canadian population, we have demonstrated that the magnitude and duration of social distancing behaviours adopted by the population during a severe pandemic can have a significant impact on the overall need for antiviral drugs. Although it is tempting to assume that in the case of a severe pandemic, informal social distancing will be enough to buffer community transmission until a safe and effective vaccine becomes available our results indicate that for a large number of scenarios, this is not a reasonable assumption. Although the cost of stockpiling antiviral treatment is substantial, relying on behaviour change to justify scaling back stockpiles is not supported by the evidence. For many scenarios simulating a severe influenza pandemic (with R0 > 1.6) and including different levels of social distancing, the demand for antivirals from a central stockpile still exceeds 20%. Stockpiling enough antivirals to treat >20% of the Canadian population as our model suggests could be the case in a severe pandemic is unrealistic from both a monetary and logistical point of view especially since there is no way to predict the probability that the next pandemic will be severe. In this case, mechanisms to decrease reliance on a finite stockpile of antivirals for treatment should focus on targeted and strategic use of antivirals in specific high risk populations and investment in novel technologies which may result in the ability to produce and distribute a safe and effective pandemic vaccine in less than 6 months. Encouraging individuals to adopt behaviours that decrease their contact rate can help to control the spread of the virus until a vaccine becomes available however; social distancing is unable to have enough of an impact to justify stockpiling fewer courses of antiviral treatment.

## Competing interests

ALG declares no competing interests.

## Pre-publication history

The pre-publication history for this paper can be accessed here:

http://www.biomedcentral.com/1471-2458/13/669/prepub
